# Factors Affecting Silica/Cellulose Nanocomposite Prepared via the Sol–Gel Technique: A Review

**DOI:** 10.3390/ma17091937

**Published:** 2024-04-23

**Authors:** Musawenkosi G. Shange, Nduduzo L. Khumalo, Samson M. Mohomane, Tshwafo E. Motaung

**Affiliations:** 1 Department of Chemistry, KwaDlangezwa Campus, University of Zululand, Empangeni 3886, South Africa; shangemg@unizulu.ac.za (M.G.S.); khumalonl@unizulu.ac.za (N.L.K.); mohomanes@unizulu.ac.za (S.M.M.); 2 Department of Chemistry, School of Science, College of Science Engineering and Technology, University of South Africa, Preller Street, Muckleneuk Ridge, P.O. Box 392, City of Tshwane 0003, South Africa

**Keywords:** cellulose, sol–gel, silica, cellulose/silica composites

## Abstract

Cellulose/silica nanocomposites, synthesised through the sol–gel technique, have garnered significant attention for their unique properties and diverse applications. The distinctive characteristics of these nanocomposites are influenced by a range of factors, including the cellulose-to-silica ratio, precursor concentration, pH, catalysts, solvent selection, temperature, processing techniques, and agitation. These variables play a pivotal role in determining the nanocomposites’ structure, morphology, and mechanical properties, facilitating tailoring for specific applications. Studies by Raabe et al. and Barud et al. demonstrated well-deposited silica nanoparticles within the interstitial spaces of cellulosic fibres, achieved through TEOS precursor hydrolysis and the subsequent condensation of hydroxyl groups on the cellulose fibre surface. The introduction of TEOS established a robust affinity between the inorganic filler and the polymer matrix, emphasising the substantial impact of TEOS concentration on the size and morphology of silica nanoparticles in the final composites. The successful functionalisation of cellulose fibres with the TEOS precursor via the sol–gel method was reported, resulting in reduced water uptake and enhanced mechanical strength due to the strong chemical interaction between silica and cellulose. In research conducted by Feng et al., the silica/cellulose composite exhibited reduced weight loss compared to the pristine cellulose matrix, with the integration of silica leading to an elevated temperature of composite degradation. Additionally, Ahmad et al. investigated the effects of silica addition to cellulose acetate (CA) and polyethylene glycol membranes, noting an increase in Young’s modulus, tensile strength, and elongation at break with silica incorporation. However, concentrations exceeding 4% (*w*/*v*) resulted in significant phase separations, leading to a decline in mechanical properties.

## 1. Introduction

Cellulose/silica nanocomposites are a type of innovative material that combines the desirable qualities of silica nanoparticles with the unique properties of cellulose, a biodegradable and renewable polymer [1]. These nanocomposites have garnered a lot of interest in recent years because of their diverse uses in disciplines like electronics, energy storage, biomedical engineering, and environmental remediation [2]. Cellulose is the most prevalent biopolymer on Earth, originating from natural sources such as wood, cotton, and other plant fibres. It has great mechanical strength, biocompatibility, and biodegradability, making it an appealing material for environmentally friendly and sustainable applications [1,3]. Pure cellulose, on the other hand, has some drawbacks, such as low thermal stability and susceptibility to moisture. Researchers have worked on integrating nanoparticles into the cellulose matrix to overcome these obstacles and improve its characteristics [4].

Silica nanoparticles, on the other hand, have a diverse set of features. Silica is an inorganic substance with exceptional mechanical strength, high thermal stability, and chemical inertness. Furthermore, silica nanoparticles have a wide surface area and are easily functionalised, allowing for better interactions with cellulose and other organic molecules. Researchers have successfully generated cellulose/silica nanocomposites that outperform pure cellulose in mechanical, thermal, optical, and barrier properties by mixing cellulose with silica nanoparticles. The nanoscale tailor ability of these nanocomposites allows for exact control over their structure and characteristics. The integration of silica nanoparticles into cellulose matrices not only improves the overall performance of the composite material but also introduces new functionalities and broadens the application possibilities [5].

Three commonly reported methods for synthesising silica nanoparticles can be found in the literature: flame synthesis, reverse emulsion, and the sol–gel method. In flame synthesis, silica nanoparticles are generated through the high-temperature decomposition of metal–organic precursors in a process often referred to as chemical vapour condensation (CVC). This entails the reaction of silicon tetrachloride (SiCl_4_) with hydrogen and oxygen. However, this method is challenging when it comes to controlling particle size, morphology, and phase composition [6,7,8].

The reverse emulsion method involves dissolving surfactant molecules in organic solvents to form spherical micelles. Nevertheless, this method is associated with high costs and difficulties in removing surfactants from the final products [9]. In contrast, the sol–gel method is widely employed to produce silica nanoparticles due to its ability to yield a pure and homogeneous product under mild conditions. This method entails the hydrolysis and condensation of metal alkoxides like tetraethyl orthosilicate (TEOS) or inorganic salts such as sodium silicate (Na_2_SiO_3_) in the presence of a catalyst like hydrochloric acid (HCl) or ammonia (NH_3_) [10,11,12].

Two models are commonly used to describe the growth mechanism of silica: monomer addition and controlled aggregation. In the monomer addition model, particle growth occurs through the addition of hydrolysed monomers to the primary particle surface. Conversely, in the aggregation model, nucleation occurs continuously throughout the reaction, resulting in the formation of primary particle aggregates that may form dimers, trimers, and larger secondary particles [7]. Both mechanisms can lead to the formation of spherical or gel network silica particles, depending on the reaction conditions. Optimisation is crucial in the sol–gel method to achieve small, homogenous, and monodispersed silica particles, with researchers finding that increasing ammonia concentration tends to increase particle size.

Drying and agglomeration are critical steps in the production of powdered silica nanoparticles. Various techniques such as freeze drying, supercritical drying, spray drying, and thermal drying are commonly used to convert liquid-phase materials into solid forms [6]. A controlled drying process leads to the formation of dispersed particles, while drying in the presence of water often results in agglomeration. The challenge lies in achieving highly dispersed nanoparticle powders, which are sensitive to processing conditions. Agglomeration behaviour can be influenced by factors like capillary drag and hydrodynamic effects during drying, and ethanol can be used as a suspension medium to reduce agglomeration. This approach has been shown to improve dispersion and reduce agglomeration, ultimately producing silica nanoparticles with enhanced properties [11].

The applications of cellulose/silica nanocomposites are numerous. They can be employed as flexible substrates, conductive films, and dielectric materials in the field of electronics. These nanocomposites show potential in energy storage applications such as lithium-ion batteries, supercapacitors, and fuel cells. Because of their biocompatibility and controlled release qualities, cellulose/silica nanocomposites have potential in drug delivery, tissue engineering, and wound healing in the biomedical area. Furthermore, these nanocomposites have the potential to be used in environmental remediation applications such as water filtration and pollutant adsorption [13].

The advantages of cellulose and silica nanoparticles are combined in cellulose/silica nanocomposites, which are an intriguing class of materials. They are extremely appealing to a variety of industries due to their special qualities and flexible applications. To further unleash the potential of these nanocomposites in tackling the issues of the modern world, ongoing research and development initiatives are exploring innovative synthesis processes, functionalisation tactics, and application fields. In this review, we aim to unpack and discuss the factors affecting the preparation of cellulose/silica nanocomposite using sol–gel method in detail [14].

## 2. Extraction of Nano Silica from Silica

**Figure 1:**
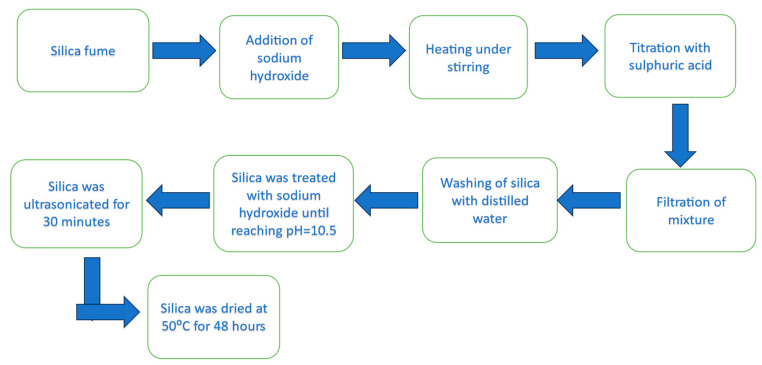
Diagrammatic representation of nano silica extraction from silica fume.

Silica is abundant in nature, existing in quartz and various living organisms. Recently, silica nanoparticles have become a subject of significant scientific and technological interest (refer to Figure 1). This heightened interest is attributed to their unique properties and structure, including a high surface area, substantial pore volume, chemical stability, low toxicity, excellent thermal characteristics, cost-effectiveness, versatility in shape transformation (from spherical to rod-like), and the capacity to encapsulate both hydrophobic and hydrophilic molecules. The research and attention surrounding this material have surged, particularly in applications related to separation and catalysis. Nano silica comes in various forms, such as rod-shaped silica, mesoporous silica, virus-like silica, and dense silica, among others. Moreover, silica nanoparticles can be tailored to exhibit specific characteristics like shape, porosity, crystallinity, particle size, and porosity, rendering them highly versatile and applicable across multiple industries. The remarkable ability of nano silica to adsorb diverse environmental pollutants is attributed to its extensive specific surface area, further elevating the interest in its potential applications [5].

**Figure 2:**
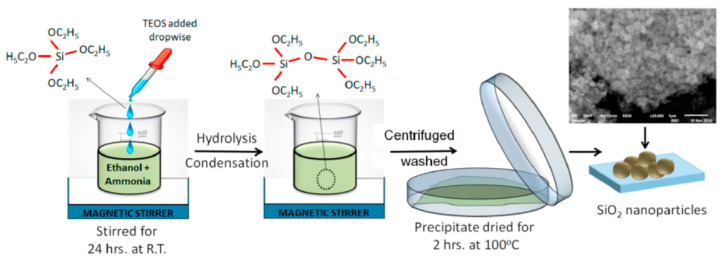
Diagrammatic representation of silica nanoparticle extraction with TOES as silica source [16].

In recent years, considerable attention from both research institutions and practical applications has been directed toward polymer/silica nanocomposites. Cellulose, as a polymer, has been employed in the synthesis and characterisation of composite materials utilising inorganic precursors such as tetramethyloxysilane (TMOS) or tetraethylorthosilicate (TEOS) (as depicted in Figure 2). The abundant hydroxyl groups inherent in cellulose, along with its extensive surface area, provide a means to functionalise the polymer’s surface and promote the adhesion of nanoparticles or biomolecules [15].

To modify cellulose pulp fibres, the sol–gel technique was employed, resulting in the incorporation of silica nanoparticles onto the fibre surfaces. The research findings indicated that while these attributes diminished proportionally with increasing fibre content, the presence of silica nanoparticles on the modified cellulose pulp fibre surfaces enhanced their resistance to axial compression and reduced the density of cement–fibre composites. This effect was attributed to the increased contact between the fibres and the cement matrix hydrates, resulting in a reduction in calcium hydroxide content and a greater presence of calcium silicate hydrates. The improved interaction between the fibres and the matrix, as well as enhanced fibre–cement performance, were made possible by the modified fibres’ reduced hydrophilicity, increased surface area, and greater surface roughness [17,18].

## 3. The Preparation of Aerogel and Xerogel via the Sol–Gel Method

The sol–gel method is a versatile technique used to prepare aerogels and xerogels, two remarkable materials known for their low density and high porosity. Aerogels are renowned for their exceptional porosity and extremely low thermal conductivity, making them suitable for applications in insulation and aerospace, and as supercapacitor materials. Xerogels, with their moderate porosity, are used in applications like catalyst supports, adsorbents, and sensors. The sol–gel method’s versatility and control over material properties make it a valuable technique for producing these unique materials. 

**Figure 3:**
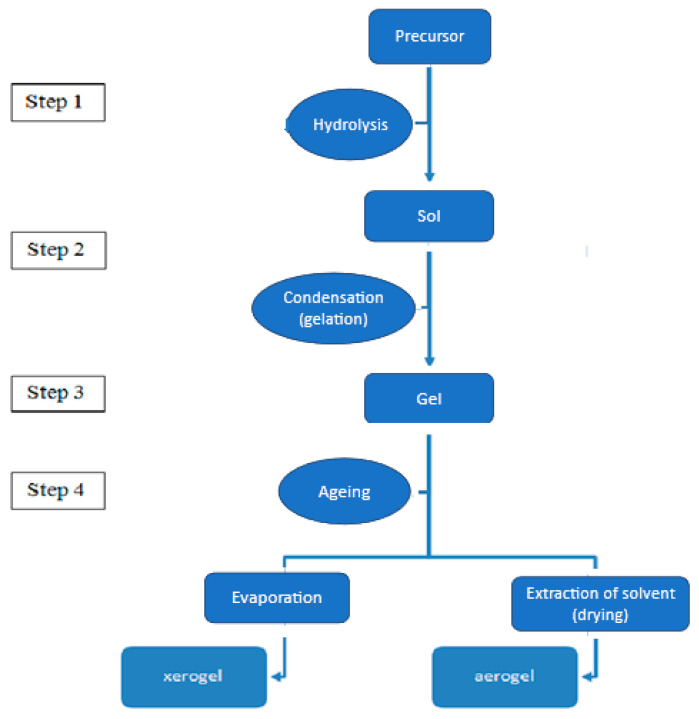
The diagrammatic representation of aerogel preparation from precursor via the sol–gel technique [25].

The sol–gel technique is a widely employed bottom-up approach utilised in the production of nanoparticles and the synthesis of inorganic–organic polymer matrices, among other applications. Through the careful monitoring of reaction parameters, this method offers precise control over factors such as particle morphology, size, and dispersion [19,20]. Notably, the sol–gel method is a versatile chemical process employed in the fabrication of glass and ceramics [21]. This process, rooted in hydrolysis and condensation reactions, facilitates the creation of pure inorganic metal oxides and inorganic–organic polymer composites [22] (as illustrated in Figure 3). Sol–gel exhibits several distinguishing characteristics, including the ability to generate high-quality nanoparticles and simultaneously produce multiple types of nanomaterials [23]. The initial stage, known as the sol, involves the suspension of particles in a liquid medium, followed by their interaction, leading to gel formation [22]. The presence of a catalyst significantly expedites the hydrolysis and condensation reactions. Sol–gel materials encompass various components, encompassing both inorganic metal oxides and organic polymers [24].

The sol–gel method is widely employed for the preparation of organic–inorganic composites due to the captivating array of morphological, mechanical, thermal, and optical properties that these materials exhibit. Through the utilisation of sol–gel, inorganic nanoparticles are uniformly distributed within the polymer matrix, forming covalent or hydrogen bonds with the polymer matrix [20]. An advantage of the sol–gel process is its simplicity and cost-effectiveness [24]. However, despite the numerous merits associated with sol–gel techniques, there are several limitations to consider, including the potential for large porosity sizes, time-consuming procedures, challenges in residue removal, the need for additional steps, significant volume shrinkage during drying, high raw material costs, and more [26].

Sol–gel finds applications in a wide range of fields, such as thin films, optical coatings, corrosion-resistant coatings, composites, and powders [27]. Parameters like the H_2_O/TEOS ratio, the choice of organic solvents, pH, the type of catalyst (base or acid catalyst), temperature, and other factors play a crucial role in determining the properties and structure of organic polymer/inorganic metal oxide hybrids when characterised through the sol–gel method [28].

## 4. Factors Affecting Cellulose/Silica Nanocomposites Prepared via the Sol–Gel Technique

The cellulose/silica nanocomposites prepared using the sol–gel technique are the subject of significant scientific interest due to their unique properties and potential applications. The performance of these nanocomposites is influenced by a multitude of factors, including the choice of precursor materials, the preparation conditions, and the processing methods employed. The type and concentration of cellulose and silica precursors, the solvent used, reaction time, and temperature all play a crucial role in determining the structure, morphology, and mechanical properties of the resulting nanocomposites. Furthermore, the addition of various additives, such as surfactants and cross-linkers, can also affect the final material’s characteristics. Understanding the interplay of these factors is essential for tailoring the properties of cellulose/silica nanocomposites for specific applications, such as in the fields of packaging, sensors, or biomedical devices. Among the important factors are the following:

- Cellulose-to-silica ratio: The cellulose-to-silica ratio in the composite can affect its overall structure and qualities. Changing the ratio can influence the interfacial interactions, dispersion, and compatibility of the two components [18,28].
- Precursor concentration: The concentration of cellulose and silica precursors in the sol–gel process can affect nanoparticle production and dispersion inside the composite matrix. It is critical to optimise the precursor concentration to achieve a homogeneous and well-dispersed nanocomposite [18].
- Acidity and pH: The pH of the sol–gel reaction system can have a considerable impact on the synthesis and characteristics of the nanocomposite. pH levels can influence cellulose solubility, silica hydrolysis and condensation rates, and the ensuing interactions between cellulose and silica nanoparticles [29].
- Catalysts and additives: A variety of catalysts and additives can be utilised to improve the sol–gel reaction while also controlling the particle size and morphology of the emerging nanoparticles. Acid or base catalysts, for example, can be used to change the pH and encourage hydrolysis and condensation reactions [20].
- Solvent selection: The solvent used in the sol–gel process has a significant impact on the solubility of cellulose and silica precursors, reaction speeds, and the creation of gel networks. The solvent must be compatible with both components and create an appropriate environment for the sol–gel reaction [28].
- Temperature and time: The temperature and time of the reaction during the sol–gel process might affect the kinetics of hydrolysis, condensation, and gelation. Controlling these factors is critical for producing the required nanocomposite particle size, dispersion, and mechanical characteristics [30].
- Surface modification approaches, such as the functionalisation or coating of cellulose and silica nanoparticles, can improve compatibility and adherence within the nanocomposite. Surface modification can improve the composite’s interfacial interactions and mechanical properties [25].
- Processing techniques: The methods used to fabricate the nanocomposite, such as casting, spin coating, or electrospinning, might have an impact on the final structure and properties. In terms of managing nanoparticle dispersion, alignment, and composite shape, each approach offers advantages and limits [28].
- Agitation: during the phase, the sol mixture in the process of gel formation must guarantee an equal production of chemical reaction in the solution, enabling all the molecules to acquire an appropriate donation of all the required chemicals to carry out the reaction properly. Gel networks typically create microscopic and macroscopic domains all over the liquid and agitation can disrupt this domain creation, allowing the network fragments to rebuild back into a larger network [31].

It is important to note that the impact of these parameters varies based on the cellulose and silica precursors used, as well as the desired qualities of the nanocomposite. To acquire the required properties of the cellulose/silica nanocomposite, these parameters are frequently optimised through rigorous testing.

### 4.1. pH Effect

One of the key factors significantly influencing the progression of reactions in the sol–gel technique is the pH level. pH plays a pivotal role in governing both the hydrolysis and condensation reactions within the sol–gel process. Furthermore, it exerts a notable impact on the morphology of the resulting metal oxide, the quantity of metal oxide generated, and the formation of metal oxide nuclei [29]. When the pH falls within the range of 2.5 to 4.5 in a sol–gel system, the reaction proceeds at its slowest rate. Altering the pH value leads to corresponding changes in the reaction rate, causing it to increase [32].

Arya et al. investigated the impact of pH on morphology, spanning from acidic to basic conditions (pH 6 to 11). The morphology of the resulting metal oxide was found to be highly dependent on the presence of H^+^ and OH^-^ ions in the sol [33]. As the pH level increased, there was an observable reduction in the crystallite size and nanoparticle size, with the particles becoming more homogeneous and spherical in shape around pH 8–9. Additionally, the maximum crystallinity was achieved at pH 9, while pH 11 led to the lowest degree of crystallinity [33].

Minju et al. focused on the effect of pH on gel formation [34]. It was observed that at pH levels below 4, gel formation required a longer time, resulting in decreased gel density and porosity exceeding 95%. Conversely, at pH 9, gelation occurred more rapidly, while at pH 10, the condensation reaction was slowed due to the dominance of sodium silicate [34].

**Figure 4:**
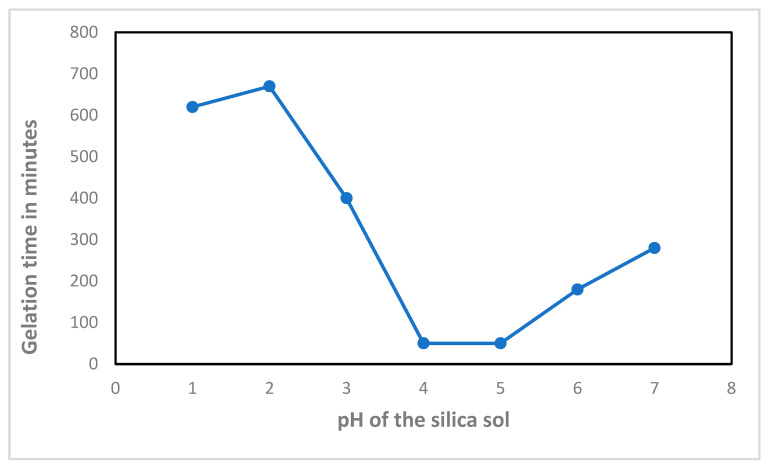
The graph indicates the relationship between the gelation time and the pH effect on silica sol [35].

Sewarande et al. delved into the impact of pH on gelation and found that gelation decreased as pH increased from 1 to 5, mainly due to pH’s ability to accelerate the condensation reaction. At pH 4, the gelation was minimal, and as pH increased from 5 to 8, both the gelation and silica condensation increased as depicted in Figure 4 [35].

Geng et al. explored the influence of different pH values on the porosity of silica aerogels. It was observed that as pH increased from 4 to 10, the porosity of the aerogels also increased, ranging from 8.68 nm to 100 nm. Notably, at pH 5, the aerogels exhibited a pore size of 10 nm, displayed more uniform characteristics, and had a closer packing structure [36].

Tzong-Horng et al. scrutinised the effect of pH (ranging from 3 to 9) on gelation and its impact on the surface area of silica. The pH level was found to be inversely proportional to the surface area of silica, with the highest surface area achieved at pH 3 and the lowest at pH 9. At pH below 3, gelation did not occur, while at pH 3, it proceeded slowly, and solid precipitation began [30]. The study also revealed that as pH increased, the silica yields also increased, reaching its maximum at pH 7. Unstable and partially redissolved gels were observed at pH values above 8, and nearly the entire gel dissolved at pH 11. The particle size and porosity increased as pH ranged from 3 to 9 [30].

Fu et al. concluded that pH significantly impacts the condensation speed and the quantity of silica nanoparticles. Silica formation occurs more easily and rapidly at higher pH values, resulting in a higher quantity of silica composites. The increased silica content led to the phenomenon of cracking. The increase in silica quantity also caused an increase in fibre skeleton thickness and a reduction in porosity, the latter being attributed to the increased bulk density caused by the growing silica aerogels. The agglomeration of silica nanoparticles caused the pore structure within cellulose to be divided, resulting in an increased surface area [37].

Salama et al. investigated the effect of methylene blue as an adsorbent in cellulose/silica nanoparticles under different pH conditions [38]. They observed that higher pH values favoured the adsorption capacity of methylene blue. At pH 7–8, a high adsorption efficiency was achieved due to ionic interactions between anionic dicarboxyl cellulose/silica and cationic methylene blue molecules. Lower pH levels led to a decrease in the adsorption sites due to higher hydronium concentration [38].

Najafi et al. explored the pH effect on particle sizes within the sol. They found that particle sizes in alkaline pH ranges were larger compared to acidic pH. pH levels below 5 resulted in very fine nanoparticles due to the lower OH- concentration in the lower pH range. As pH increased, the particle sizes also increased due to a higher OH- concentration, leading to increased hydrolysis [39].

Zhong et al. examined the effect of pH on the relationship between solid and liquid interactions. The pH of the dye solution was crucial for adsorption capacity. They observed a proportional relationship between the adsorption of methylene blue and pH, with pH 5 exhibiting 93.6% adsorption. The adsorption of cellulose/silica composite was 6.241 mg/g. At higher pH values, adsorption remained relatively unchanged [40].

Surtalie et al. investigated the pH effect on glucose concentration after hybrid cellulose hydrolysis. They found that the most favourable pH for both cellulose/silica nanocomposite and pure cellulase was pH 4.8 [41].

Milea et al. and Fardad et al. pointed out that both the hydrolysis and condensation reactions, which are usually regulated by the pH of the solution, are crucial for the microstructure of the metal oxide manufactured through the sol–gel method [42,43]. Hydrolysis kinetics are favoured under acid-catalysed conditions, while condensation typically begins after the completion of hydrolysis. Basic-catalysed reactions lead to faster condensation than hydrolysis, resulting in highly condensed species that may aggregate into fine particles. To enhance the hydrolysis and condensation reactions, acid or alkaline catalysts can be used to adjust the pH of silicon alkoxides, which simplifies the alteration of the surface charge. An isoelectric point of pH 2.2 helps stabilise the pH of silica, preventing aggregation and agglomeration due to reduced particle-to-particle interactions. Gelation occurs when the distance between particles is reduced, but precipitation occurs when particles become too large. Agglomeration in the sol–gel process, based on strong oxides, is irreversible, particularly after drying.

Karakuzu et al. examined the effect of pH on porosity and surface area under the influence of citric and nitric acid as shown in Table 1 [44]. The results indicated that the porosity of silica aerogels increased with higher pH values for both nitric and citric acid, but at higher pH, the aerogel density was minimised.

In summary, pH plays a crucial role in various aspects of sol–gel processes, including morphology, gel formation, condensation reactions, surface area, adsorption capacity, and other characteristics, depending on the specific context and materials involved. The pH level can be adjusted to tailor the properties of the resulting materials for specific applications.

### 4.2. Catalyst

The sol–gel technique can be conducted using either acid-catalysed or base-catalysed reactions. Typically, HCl is the acid catalyst of choice, while ammonia is commonly used as the base catalyst. Other catalysts, including fluorides, can also be employed. Nawaz et al. investigated the impact of acid and base catalysts on silica aerogels and found that samples prepared with a base catalyst displayed larger average pore sizes and smaller mass diffusivity, while samples prepared with an acid catalyst exhibited smaller pore sizes and higher mass diffusivity values [45].

Rao et al. explored the influence of various catalysts and solvents on the physical properties and monolithicity of materials produced using the sol–gel technique [46]. They observed that strong bases and solvents with longer chain lengths resulted in semi-transparent aerogels, while a strong acid–weak base mixture produced transparent aerogels with cracks. A weak acid–weak base catalyst mixture produced shrunken, semi-transparent aerogels, and a weak base catalyst with a shorter chain length solvent yielded high-quality aerogels with a large surface area, low density, and refractive index while maintaining transparency.

Sequeira et al. studied the effect of different mineral acids (HCl, HNO_3_, H_3_PO_4_, and H_2_SO_4_) and various heteropoly acids as catalysts in cellulose/silica nanocomposites [19]. Heteropoly acids exhibited thermal stability between 350–400 °C and displayed a greater affinity for silica than cellulose polymer, unlike strong mineral acids. Among the mineral acids, nitric acid proved to be the most effective, despite its weaker nature compared to hydrochloric and sulfuric acid.

Milea et al. and Gurav et al. emphasised the influence of the catalyst used in the precursor solution on nanocomposite thickness, optical quality, shrinkage, and porosity [42,47]. H_2_SO_4_ and H_3_PO_4_ were found to accelerate the hydrolysis of TEOS in ethanol, producing particles with a higher concentration of hydroxyl groups in the solution. This, in turn, led to a condensation reaction that produced nanocomposites with lower porosity. However, the poor quality of films resulting from H_2_SO_4_ and H_3_PO_4_ catalysts made them less desirable for various applications. These catalysts led to the precipitation of large particles and the formation of films.

Jyoti et al. examined the effect of NH_4_F concentration on the properties of aerogels when maintaining specific molar ratios in the sol–gel process [47]. The addition of NH_4_F led to simultaneous hydrolysis and condensation reactions in the one-step sol–gel process, with F ions catalysing the displacement of -OC_2_H_5_ groups through a bimolecular nucleophilic mechanism. This catalytic effect sped up the gelation process, making it more pronounced than that influenced by hydroxyl ions. The one-step sol–gel process resulted in transparent, hydrophobic, and low-density silica aerogels.

**Figure 5:**
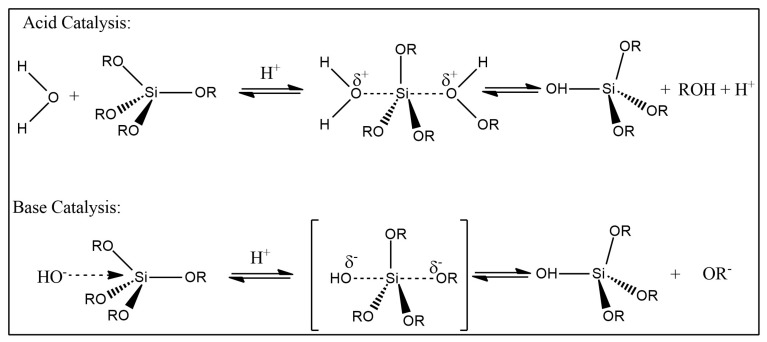
The reaction of a base catalyst and acid catalyst with silica [48].

Furthermore, the concentration of NH_4_F was varied in a two-step sol–gel process to observe its effects on bulk density and gelation time. Increasing NH_4_F concentration led to a faster condensation rate, reducing gelation time. The two-step sol–gel process was found to produce higher-quality aerogels compared to the one-step method. A mixed catalyst system, combining NH_4_OH and NH_4_F in the second step, was employed to enhance aerogel quality and optical transmission. In varying the volume of NH_4_OH, low-density (0.065 g/cm^3^), superhydrophobic (153.8), thermally stable (380 °C), and highly optically transparent (95%) aerogels were obtained at an NH_4_OH volume of 0.5 mL. This demonstrates the potential to improve the quality of aerogels through a mixed catalyst system [47]. General mechanism for both acidic and basic catalysis is summarised in Figure 5 [48].

### 4.3. Acid Catalyst

The sol–gel technique allows for the use of various acids, including hydrochloric acid, sulfuric acid, oxalic acid, nitric acid, and acetic acid. Hydrolysis is generally favoured, and in the first step, it proceeds rapidly while condensation is limited. During this initial step, an open network structure is formed, leading to the condensation of small clusters. The acid catalyst accelerates the hydrolysis of TEOS and results in the formation of a loosely interconnected polymer-like structure. The initial hydrolysis phase, where the precursor material is converted to trialkoxy silanol groups, occurs more rapidly than the secondary hydrolysis phase due to the unfavourable protonation of the silanol group. This leads to the formation of a transparent nanocomposite with morphology sizes below 100 nm, making acid catalysts preferred for the preparation of polymer/silica nanocomposites [24].

Sequeira et al. demonstrated that an increase in the refractive index is associated with a decrease in thickness, pore volume, and shrinkage over time [20]. Acid-catalysed syntheses typically employ strong mineral acids, which can weaken or damage the acid-labile host polymer matrix during the drying and ageing processes. These mineral acids, particularly those that are thermally unstable and volatile, can also cause corrosion issues in equipment throughout the material production process and pose conditional concerns that limit the final use of the material. In this context, the use of potent but non-thermally stable and volatile solid acids, such as heteropoly acids (HPAs), in sol–gel syntheses warrants further study [20].

### 4.4. Base Catalyst

When using a basic catalyst, faster condensation and slower hydrolysis are observed, resulting in more compact colloidal particles. The basic catalyst typically yields a non-transparent composite with phase dimensions exceeding 100 nm [24]. Rao et al. investigated the effect of base catalysts with varying concentrations on gelation time [46]. They observed that as the concentration of the base catalyst increased from 0 to 3 M, the gelation time decreased from 10 to 2 min due to increased condensation. In the absence of ammonium hydroxide as a catalyst, the alcohols catalysed by the acid exhibited longer gelation times. As the base concentration increased from 0 to 1 M, a decrease in bulk density was observed, and a slight increase occurred as the base catalyst concentration exceeded 1 M. An increase in the base catalyst concentration to 1 M resulted in larger particle size and increased particle connectivity. However, with base catalyst concentrations above 1 M, the condensation rate became too rapid, resulting in smaller particle sizes and decreased connectivity between particles, leading to aerogel shrinkage [46].

Arora et al. examined the effect of ammonia catalyst concentration on the size of nanoparticles [49]. They found that nanoparticle size decreased with a decrease in ammonia concentration, as reduced ammonia concentration led to decreased condensation, prolonging the nucleation period. This resulted in more nuclei formation and the formation of smaller nanoparticles. Norazmi et al. studied the effect of ammonium hydroxide catalyst on silica nanoparticles prepared using the sol–gel technique [50] as shown in Table 2. The volume of ammonium hydroxide played a crucial role in the size and distribution of silica nanoparticles. Ammonium hydroxide acted as a catalyst for both the hydrolysis and condensation of TEOS. Decreasing the volume of ammonium hydroxide led to a reduced weight ratio, resulting in smaller-sized silica nanoparticles. Higher volumes of ammonium hydroxide produced larger average-sized silica nanoparticles due to controlled aggregation.

### 4.5. Temperature

**Figure 6:**
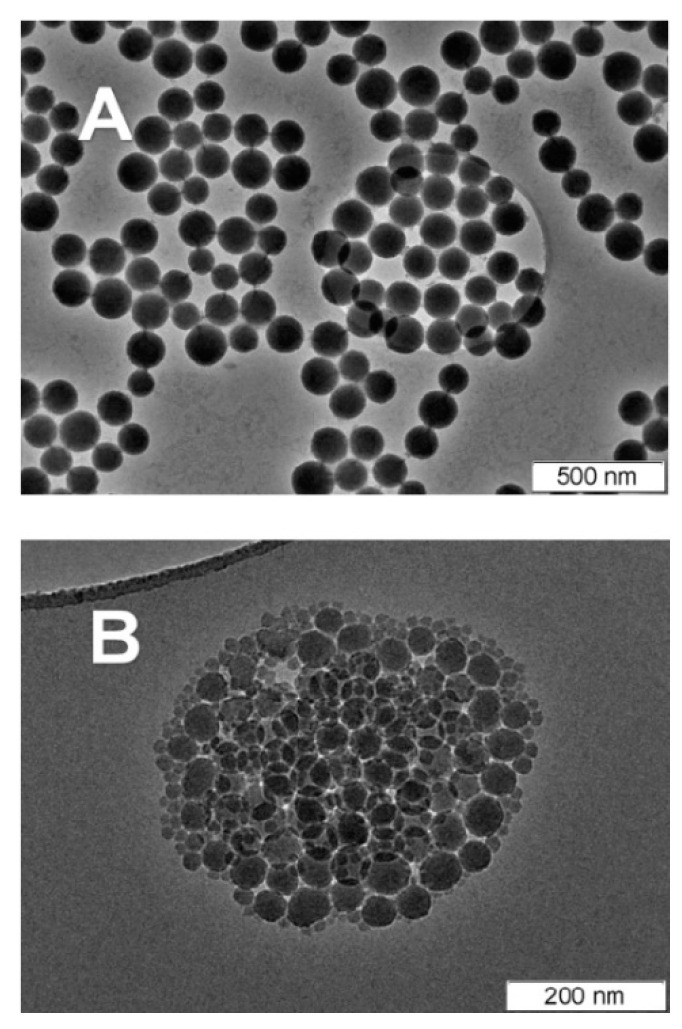
The TEM images representing silica nanoparticles prepared at (**A**) 50 °C and (**B**) 70 °C.

Twej et al. investigated the influence of temperature on the gelation of cellulose/silica nanocomposites [50]. Temperature is one of the key factors affecting reaction rates. As the temperature increases, agglomeration becomes more prominent, with faster agglomeration occurring at 55 °C compared to 40 °C and 25 °C (as shown in Figure 6). This temperature effect is attributed to the increased speed of particles at higher temperatures, leading to more collisions. Higher temperatures also reduce gelation time and result in an increased number of secondary particles formed.

Zhong et al. examined the temperature effect on the adsorption of methylene blue on cellulose/silica hybrid [40]. They varied the temperature from 15 to 75 °C while keeping other parameters such as pH, dose, contact time, volume, and initial CMB constant. It was observed that increasing the temperature from 15 to 35 °C resulted in increased adsorption. This rise in temperature accelerated the adsorption rate, leading to enhanced molecular motion and increased methylene blue adsorption by cellulose/silica. However, further increases in temperature led to a decrease in adsorption capacity, with an exothermic process occurring at higher temperatures, causing the absorbed methylene blue to be released. The highest adsorption capacity was observed at 35 °C.

Sutarlie et al. studied the temperature effect on cellulose and cellulose/silica hybrids [41]. They found that temperatures ranging from 25 °C to 50 °C did not denature cellulose and cellulose/silica nanocomposite. However, at 51–60 °C, a 14% decrease in cellulose/silica nanocomposite was observed, while cellulose exhibited a 28% decrease. This indicates greater stability in cellulose/silica compared to a free cellulose polymer.

Fonseca et al. observed that an increase in temperature led to a higher reaction rate, resulting in the increased entrapment of water and alcohol molecules during the preparation of silica, leading to the formation of larger volume pores [51]. Tzong-Horng Liou investigated the effect of ageing temperature on the surface area of silica produced at pH 7 [30]. They observed that increasing the temperature from 20 °C to 50 °C led to an increase in surface area due to faster silica growth. However, a further increase in temperature from 50 to 120 °C resulted in a decrease in surface area due to a faster gelation rate at high temperatures.

Lazareva et al. investigated the temperature effect on silica synthesised via sol–gel [52]. They maintained the initial variables (TEOS, NH_3_, water, and ethanol) and increased the temperature from 50 °C to 70 °C. It was observed that the silica nanoparticles decreased in size by a factor of 3, ranging from 150–100 nm to 50–30 nm. Higher temperatures, coupled with increased water and catalyst concentrations, led to the formation of non-uniform silica nanoparticles due to faster condensation and hydrolysis. Lower temperatures resulted in larger particle sizes and the precipitation of silica nanoparticles.

Betiha et al. investigated the temperature effect on gel strength under different time intervals (10 s and 10 min) [53]. They found that increasing the temperature led to a decrease in gel strength at both the 10 s and 10 min intervals.

Verma et al. explored the temperature effect on the synthesis of silica nanoparticles, increasing the temperature from 78 °C to 650 °C [54]. They observed that as the temperature increased from 78 °C to 650 °C, the size of the silica nanoparticles decreased from 29 nm to 85 nm.

Surtalie et al. examined the optimum temperature effect on glucose concentration after the hydrolysis of cellulose [41]. The cellulose/silica nanocomposite exhibited a higher optimum temperature compared to pure cellulose, with the hybrid cellulose aggregate showing an optimum temperature of 51 °C, while pure cellulose had an optimum temperature close to 49 °C. Increasing the temperature from 50 to 60 °C resulted in a 14% reduction in the cellulose/silica nanocomposite and a 28% reduction in pure cellulose. This highlights the greater stability of cellulose/silica composites compared to pure cellulose.

Rao et al. studied the temperature effect on highly pure silica catalysed by citric acid and TEOS through heating in air at around 98.0 °C for 8 h [55]. An initial temperature increase from 25 to 250 °C led to a decrease in the bulk density of aerogels (from 0.22 × 10^3^ to 0.19 × 10^3^ kg m^−3^), primarily due to weight loss while the volume and porosity remained constant. A further temperature increase above 250 °C resulted in increased weight loss and volume shrinkage, with a decrease in porosity at temperatures above 250 °C. When the temperature reached 980 °C, xerogels were produced as a transparent glass mass with no porosity.

Milea et al. emphasised that although the role of chemistry in processes after gelation may not seem particularly significant, it plays a crucial role in aspects such as the homogeneity, purity, and porosity of silica gels [42]. During the drying process, the loss of water, alcohol, and other volatile substances leads to gel contraction and increased structural tightness. This can make it challenging to obtain monoliths due to the risk of fractures if strains cannot be relaxed. Drying additives like non-hydrolysing organic groups (methyl or phenyl) can be used to relieve this pressure, allowing structural relaxation and reducing the risk of increased material porosity. The addition of a catalyst to the gelation mixture may accelerate the ageing process and produce materials with stable properties suitable for practical applications.

### 4.6. Concentration

Betiha et al. examined the impact of concentration on gel strength under different time intervals (10 s and 10 min) [53]. They found that an increase in concentration led to higher gel strength in both time intervals.

Guangbao et al. investigated the effect of sodium silicate concentration on the formation of silica sol [56]. They observed that concentration had a significant influence on particle size and surface area due to nucleation and growth reactions. At low concentrations and temperatures, the surface area of silica nanoparticles increased until reaching a maximum. However, as the silicate concentration increased, more cores formed, leading to an increase in surface area. At higher silicate concentrations, gelation increased, resulting in a reduction in pore diameter.

Vajihe et al. studied the effect of sodium hydroxide concentration on the synthesis of silica nanoparticles. The sodium hydroxide concentration varied between 1M and 3M at 80 °C for 30 min [57]. An increase in sodium hydroxide concentration resulted in a steep increase in silica dissolution efficiency. The concentration of 2.5M sodium hydroxide exhibited a maximum dissolution efficiency of 91%.

Kim et al. explored the impact of solvent and catalyst concentration on the size of silica in the preparation of cellulose nanofibres/silica nanocomposite [58]. They found that reducing the concentration of ammonium hydroxide resulted in smaller silica nanoparticles and a decrease in silica content to approximately 67.81 wt%.

Sai et al. investigated the flexibility of the cellulose/silica aerogel network in 10 runs [59]. They observed that increasing the concentration of TEOS led to a higher bulk density of aerogels, increased surface area, and decreased porosity of the aerogels. Another good example is presented in Table 3 [60]. 

Hernandez et al. emphasised that while it is well known that TEOS polymerisation in acidic conditions produces small, stable particles, the structure of the resulting materials is heavily dependent on the initial concentration of TEOS, even when other important parameters are held constant [18]. They used AFM in tapping mode to analyse the morphology of sol–gel-produced polymer/silica nanocomposites.

### 4.7. Effect of H_2_O/TEOS

**Figure 7:**
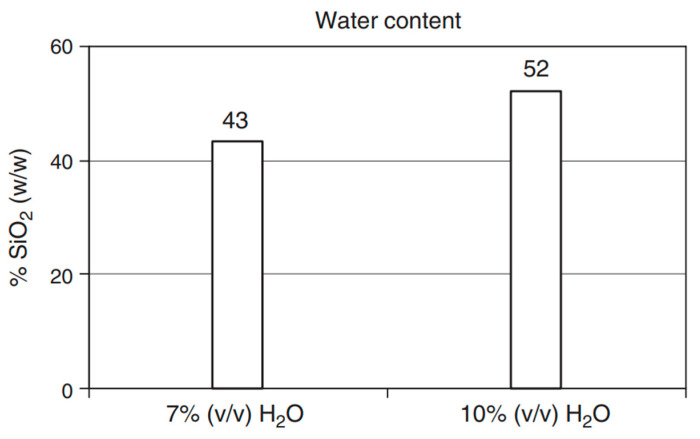
The diagrammatic representation of water influence on cellulose/silica hydrogel. Reaction conditions: 24 h, 20 °C, TEOS/EtOH/H_2_O solution 20:45:5 (*v*/*v*/*v*) (H_2_O/TEOS = 3.2 mol/mol) and 20:43:7 cm^3^ (H_2_O/TEOS = 4.4 mol/mol), PW12 concentration of 3.0 × 10^−4^ mol/L [20].

The available information indicates a limited focus on the effect of the water-to-nano silica ratio. Most researchers have concentrated on the H_2_O/TEOS ratio, with TEOS serving as the source of nano silica. Sequeira et al. conducted a study to explore the impact of tetraethyl orthosilicate (TEOS) and water on the synthesis of cellulose/silica composites via the sol–gel process, using heteropoly acids (HPAs) as catalysts [20] (Figure 7). The study revealed that water molecules had a significant influence on the rate of TEOS hydrolysis, affecting both the hydrolysis and condensation reactions. Increasing the water content from 3.2 to 4.4 mol H_2_O/mol TEOS (equivalent to 7% and 10% *w*/*w*, respectively) promoted the adsorption of silica nanoparticles by cellulose fibres. This effect may be attributed to the increased molecular weight of siloxane polymers resulting from the enhanced alkoxysilane condensation reaction in the presence of higher water molar proportions in the reaction system.

Sai et al. used a factorial design to examine how preparation conditions affected the textural and structural characteristics of NH_3_-catalysed silica xerogels [59]. Their study showed that the concentration of chemicals and temperature had no effect on the structural bonds in the siloxane microstructure. However, the water/TEOS molar ratio had a significant impact on various properties, including specific surface area, pore volume, and average pore size. Specifically, an increase in the water/TEOS molar ratio led to higher specific surface area, pore volume, and average pore size.

McDonagh et al. investigated the impact of the water-to-precursor ratio (R) on film thickness using the sol–gel technique, recognising that water plays a significant role in both hydrolysis and condensation reactions [61]. The study considered various R values, such as 2, 4, 5, and 6. Under the conditions of sol fabrication at pH 1, immersion at 1 mm, and ageing for 5 h at 70 °C, it was observed that increasing R values resulted in an increase in film thickness. This increase in film thickness can be attributed to the faster hydrolysis at pH 1 compared to condensation, and the additional water introduced by higher R values promoted the hydrolysis process. It was also noted that the impact of R values on hydrolysis was less pronounced at higher pH values. At larger R values and low pH, the gel time decreased, resulting in thicker films.

Cai et al. investigated the effect of the quantity of tetraethyl orthosilicate (TEOS) on the porosity and pore diameter of silica nanoparticles in cellulose/silica nanocomposites [62]. They found that increasing the quantity of added TEOS resulted in smaller silica nanoparticles, reduced porosity, and a smaller pore diameter as well as a decrease in the BET surface area.

Esposito et al. conducted experiments to explore the impact of the H_2_O/TEOS ratio (Rw) on the preparation of porous silica [63]. They compared traditional sol–gel methods with an alcohol-free technique and observed that the H_2_O/TEOS ratios significantly influenced the gel properties. The use of an alcohol-free approach resulted in changes in gel properties, including increased surface area and average pore diameter. The volume of micropores became insignificant in the alcohol-free preparation.

Fardad et al. investigated the effect of the molar ratio of water to TEOS (R) on film thickness, porosity, and shrinkage [64]. They found that as the R value increased, film thickness, shrinkage, and pore volume decreased, whereas the refractive index increased. The changes in indices and porosities with respect to thermal treatment temperature became more gradual with increasing R. This is in line with previous research that suggested that increasing R results in more thorough hydrolysis and increased density, as well as lower sol viscosity due to increased dissolution.

### 4.8. Evaluation of the Effects of Factors Affecting Silica/Cellulose Nanocomposite Prepared via the Sol–Gel Technique on Applications of the Nanocomposites

Silica/cellulose nanocomposites, prepared through the sol–gel technique, have garnered significant interest in recent years due to their unique combination of properties derived from both silica nanoparticles and cellulose fibres. The preparation process involves intricate control over various factors, such as precursor concentrations, pH, temperature, and processing methods. These factors significantly influence the final nanocomposite’s structure, morphology, and properties, subsequently impacting their applications. This critical evaluation delves into the effects of these factors on the applications of silica/cellulose nanocomposites, shedding light on their potential and limitations in various fields.

#### 4.8.1. Structural and Morphological Impact

The control of factors like precursor concentrations and pH during the sol–gel synthesis plays a pivotal role in determining the nanocomposite’s structure and morphology. A well-defined structure and uniform dispersion of silica nanoparticles within the cellulose matrix enhance mechanical strength, thermal stability, and barrier properties. Deviations in these factors can lead to agglomeration or uneven distribution, severely compromising the mechanical integrity and overall performance of the nanocomposites. Morphological studies play a crucial role in understanding the structure and performance of silica/cellulose composites. Achieving the desired membrane morphology is essential for optimising performance in specific applications. Neto and colleagues [65] conducted a study on silica/cellulose nanocomposites using two different methods: the layer-by-layer deposition of silica nanoparticles on cellulose fibres and the in situ synthesis of silica in the presence of cellulose fibres. The choice of synthesis method had a significant impact on the morphology of the resulting nanocomposites. The layer-by-layer approach produced well-defined silica nanoparticles that were evenly distributed on the cellulose fibre surface, thanks to a balanced interaction between the silica coating and polyelectrolytes. In contrast, in situ synthesis led to homogeneously coated silica/cellulose nanocomposites due to the condensation of silica oligomers during growth and ammonia concentration, which dispersed the silica particles for uniform adsorption.

Studies by Reddy et al. [66] and Song et al. [67] revealed that fractured surfaces of cellulose/silica composites were considerably rougher compared to neat cellulose. Morphological examinations indicated that silica nanoparticles were uniformly dispersed without aggregation, signifying good interfacial interaction. However, composite films with 5 wt% nano silica showed rough surfaces with some nano silica aggregation. Similarly, Arthanareeswaran et al. [68] modified the morphological structure of cellulose to enhance membrane performance by incorporating nano silica. SEM results showed that increasing SiO_2_ content up to 40 wt% resulted in more pores and increased pore density on the membrane’s top surface. Ashori et al. [69] also reported strong interfacial adhesion between bacterial cellulose fibres and nano silica particles without noticeable aggregates observed through SEM.

**Figure 8:**
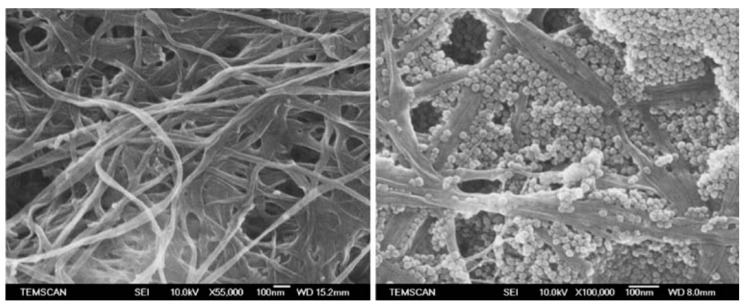
SEM representing the modification of cellulose fibres (**left**) and silica/cellulose nanocomposite (**right**) [71].

Raabe et al. [70] and Barud et al. [71] conducted morphological analyses and found well-deposited silica nanoparticles embedded in the interstitial spaces of cellulosic fibres (refer to Figure 8). This was achieved through the hydrolysis of the TEOS precursor, followed by the condensation of hydroxyl groups on the cellulose fibre surface. The addition of TEOS led to a strong affinity between the inorganic filler and the polymer matrix, emphasising the significant effect of TEOS concentration on the size and morphology of silica nanoparticles in the final composites. Furthermore, the successful functionalisation of cellulose fibres with TEOS precursor via the sol–gel method was reported, resulting in reduced water uptake and improved mechanical strength due to the strong chemical interaction between silica and cellulose.

Meer et al. [72] investigated the impact of pH value on the condensation rate and silica nanoparticle deposition on the CNF (cellulose nanofibre) matrix through sol–gel synthesis using TEOS as a precursor. Higher pH values facilitated the faster formation of silica nanoparticles within the matrix. Similar findings were reported by Ghiorghita et al. [73] in a study of a novel silica/polyelectrolyte multilayer core–shell composite, with a higher polymer concentration resulting in more deposited polycation. Selakjani et al. [74] examined the effect of silica in a cellulose nanocomposite, revealing well-dispersed spherical silica nanoparticles on the cellulose fibre surface due to the micelle effect of a surfactant, ensuring good particle dispersion during the doping process.

#### 4.8.2. Mechanical Properties

Silica/cellulose nanocomposites exhibit promising mechanical properties, including enhanced tensile strength and modulus. The homogeneity in nanoparticle dispersion, influenced by factors such as stirring speed and processing time, is crucial. Insufficient mixing or prolonged processing can result in weak interfacial interactions, limiting the load transfer between the matrix and nanoparticles. Consequently, this compromises the nanocomposites’ mechanical properties, restricting their applicability in high-stress environments. Reddy and colleagues [66] investigated the mechanical characteristics of regenerated cellulose that had been reinforced with silica nanoparticles. The findings revealed a notable increase in tensile strength and modulus when the silica concentration was low. This enhancement in mechanical properties was attributed to the effective dispersion of silica within the regenerated cellulose matrix. However, at higher silica concentrations, the particles tended to agglomerate within the polymer matrix, which had a detrimental impact on the mechanical properties. Similar observations were made by Song et al. [67], Ashori et al. [69], and Xie et al. [75] in their respective studies on various nanocomposite materials reinforced with silica.

**Figure 9:**
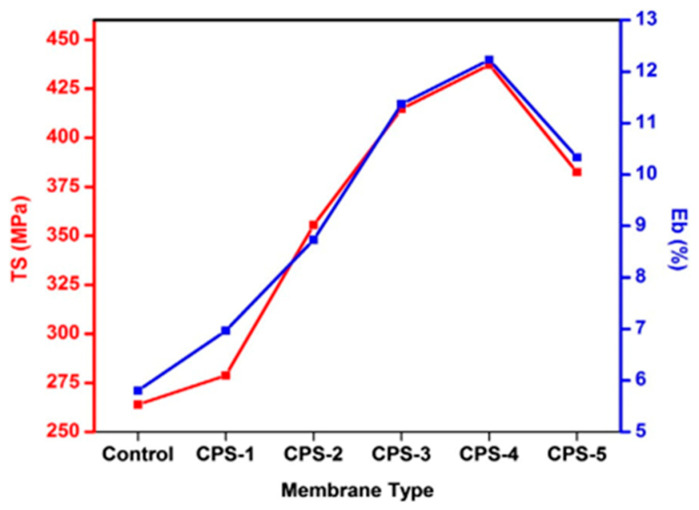
Tensile strength and the extent of elongation at the point of rupture for both the unaltered and adjusted membrane [76].

Arthanareeswaran and colleagues [68] examined the influence of silica particles on cellulose acetate (CA) blend membranes, covering a range of silica contents from 0 to 40% by weight. Initially, the mechanical properties (tensile strength, tensile stress, and elongation at break) of the CA/silica composites showed improvement. However, as the silica content exceeded 10 wt%, these properties began to decline. This decline was associated with the agglomeration of silica, which led to the suppression of micro voids and weakened interactions between the polymer and the inorganic filler. These findings align with those reported by Ahmad et al. [76], who investigated the impact of silica addition on the mechanical properties of membranes comprising CA and polyethylene glycol (Figure 9). Their results demonstrated an increase in Young’s modulus, tensile strength, and elongation at break with the incorporation of silica nanoparticles, as compared to the neat CA/polyethylene glycol membranes. However, when the silica concentration exceeded 4% (*w*/*v*), large phase separations occurred due to an excessive silica concentration, resulting in a decline in mechanical properties.

Wu and collaborators [77] investigated the mechanical properties of cellulose/silica aerogel nanocomposites through compression stress and strain tests. Their results indicated that aerogels with higher silica content (79%) exhibited cracking at low strain levels (20%) when subjected to increased external pressure. In a separate study by Wojciechowska et al. [78], the nanocomposites of CA butyrate and TEOS prepared using the sol–gel method demonstrated superior mechanical properties compared to unmodified CA butyrate. These improvements were attributed to enhanced interfacial adhesion and increased efficiency in the stress transfer mechanism between the two components. Notably, the unaltered CA butyrate displayed the highest elongation at break, showing a 28% increase compared to the modified CA butyrate/silica nanocomposites. Finally, a study by Ibrahim et al. [79] reported an enhancement in mechanical properties (burst, short span, and tensile strength) at low silica concentrations. However, when the silica concentration exceeded 4 wt%, roughness and elongation at break decreased due to the agglomeration of silica nanoparticles in specific regions within the hybrid material. 

#### 4.8.3. Thermal Stability

**Figure 10:**
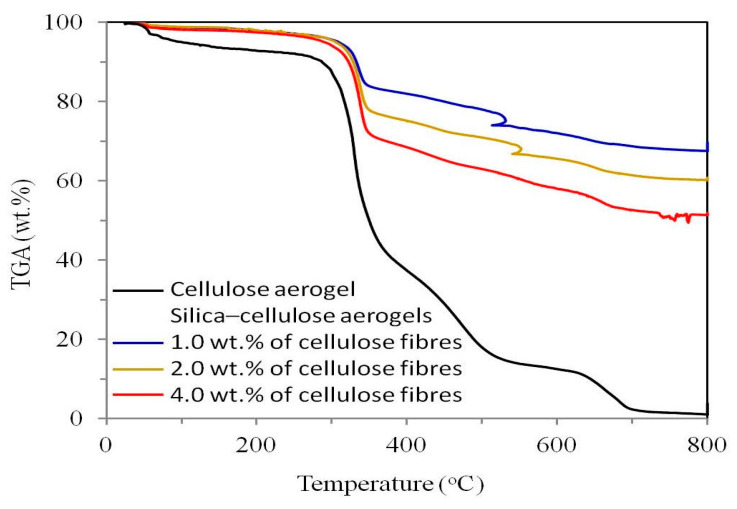
The thermal gravimetric analysis (TGA) curve of the silica/cellulose aerogel is compared to that of the pure cellulose matrix. These aerogels, composed of silica and cellulose, were produced from initial suspensions of the cellulose matrix containing different concentrations of cellulose fibres, namely 1%, 2%, and 4% by weight [80].

The thermal stability of nanocomposites, vital for applications in high-temperature environments, is affected by factors like calcination temperature and annealing duration. Proper control ensures the removal of organic components and enhances the thermal stability of the nanocomposite. Inadequate control might lead to residual organic content, diminishing the material’s stability and limiting its utility in applications requiring high-temperature resistance. As per the available literature, the introduction of silica nanoparticles into cellulose nanocomposites is linked to an enhancement in their thermal stability. In a study conducted by Feng and colleagues [80], it was observed that the silica/cellulose composite exhibited reduced weight loss compared to the pristine cellulose matrix (Figure 10). The inclusion of silica within the cellulose structure led to an elevation in the temperature at which composite degradation occurred. Similarly, Xie et al. [75] reported an enhancement in the thermal properties of cellulose hybrid composites when silica was incorporated. The thermal analysis of cellulose/silica nanocomposites revealed a progression from larger to smaller endothermic peaks with increasing silica content, indicating a substantial interaction between silica and cellulose. This phenomenon suggests that organic–inorganic nanocomposites exhibit not only the thermal characteristics of the inorganic constituents but also those of the organic polymer.

Sheykhnazari and associates [81] conducted a study involving bacterial cellulose (BC) composites enriched with silica nanoparticles. The results from the thermogravimetric analysis (TGA) demonstrated that all BC/silica composites exhibited greater thermal stability compared to pure BC. Notably, the composite containing 3 wt% silica displayed robust thermal stability, while the sample with 7 wt% silica exhibited a higher degradation temperature in comparison to pure BC and other composite materials.

Furthermore, Raabe et al. [70] investigated the influence of reaction parameters on the deposition of silica nanoparticles onto cellulose fibres. The findings revealed that silica nanoparticles bonded effectively to the surface of the cellulose matrix, resulting in an enhancement in the thermal stability of the composites and an increase in the temperature at which degradation commenced.

#### 4.8.4. Barrier Properties

Silica/cellulose nanocomposites find applications in packaging materials and coatings due to their excellent barrier properties against gases and liquids. Factors influencing porosity and surface area, such as drying techniques and precursor ratios, directly impact these barrier properties. Insufficient control may result in inadequate barrier performance, reducing the effectiveness of these nanocomposites in applications demanding stringent containment properties.

#### 4.8.5. Biocompatibility and Environmental Impact

The biocompatibility of silica/cellulose nanocomposites is essential for biomedical applications and eco-friendly products. Factors affecting surface modification and particle size distribution play a crucial role. Inadequate modification or particle size control might lead to toxic effects, limiting their application in biomedicine. Additionally, the environmental impact of the synthesis process, influenced by precursor choice and waste disposal methods, is a significant concern. Sustainable practices need to be employed to minimise the environmental footprint of these nanocomposites.

Silica/cellulose nanocomposites prepared via the sol–gel technique exhibit immense potential in various applications, ranging from packaging materials to biomedical devices. However, the critical evaluation of factors influencing their synthesis and properties is imperative to unlock their full potential. Careful control of precursor concentrations, pH, processing methods, and surface modifications is necessary to ensure the uniform dispersion of silica nanoparticles within the cellulose matrix. This control directly impacts structural integrity, mechanical properties, thermal stability, barrier performance, biocompatibility, and environmental sustainability. As research continues to refine the synthesis process and enhance the understanding of these factors, silica/cellulose nanocomposites can be tailored to meet specific application requirements, paving the way for innovative and sustainable solutions in diverse fields.

## 5. Conclusions

In conclusion, the sol–gel synthesis of cellulose/silica nanocomposites is a complex process influenced by key factors that are essential for tailoring the properties of the resulting materials for various applications. 

The cellulose-to-silica ratio and precursor concentration play fundamental roles in shaping the nanocomposite’s structure and characteristics, with adjustments needed for uniformity. Acidity, pH, and the choice of catalyst (acid or base) influence hydrolysis and condensation reactions, thereby affecting material morphology and nanoparticle size. 

Solvent selection is critical, impacting precursor solubility and reaction kinetics. Temperature and reaction time control hydrolysis, condensation, and gelation kinetics, which are vital for achieving the desired particle size and dispersion. 

Surface modification techniques and processing methods enhance the overall performance of nanocomposites, and agitation is crucial during gel formation for proper chemical reactions. 

The water-to-nano silica ratio, often overlooked, is significant for hydrolysis, condensation, and structural characteristics, providing insights for tailoring nanocomposite properties. 

pH levels can be adjusted effectively to tailor nanocomposite properties for specific applications, impacting morphology, crystallinity, and porosity. 

The choice of catalyst (acid or base) significantly impacts material properties, with acid catalysts yielding transparent nanocomposites suitable for polymer/silica applications, and base catalysts producing non-transparent composites with larger particles. Catalyst concentration and type further influence material characteristics. 

Temperature control is crucial for achieving the desired properties, impacting the kinetics of reactions and nanoparticle size. 

These factors play a crucial role in shaping the structure, morphology, and mechanical characteristics of nanocomposites, allowing for tailored applications. Previous studies have illustrated the effective deposition of silica nanoparticles within the interstitial spaces of cellulosic fibres through the hydrolysis of the TEOS precursor, followed by the condensation of hydroxyl groups on the cellulose fibre surface. The successful functionalisation of cellulose fibres with the TEOS precursor via the sol–gel method was documented, resulting in reduced water uptake and increased mechanical strength due to the robust chemical interaction between silica and cellulose. 

Silica/cellulose composites exhibit decreased weight loss compared to the pristine cellulose matrix, and the incorporation of silica raised the temperature of composite degradation. Additionally, the effects of silica addition to cellulose acetate (CA) and polyethylene glycol membranes were observed, showing an increase in Young’s modulus, tensile strength, and elongation at break with silica incorporation. However, concentrations exceeding 4% (*w*/*v*) led to significant phase separations, resulting in a decline in mechanical properties. Continuous research aims to unlock the full potential of these materials in addressing modern challenges.

## Figures and Tables

**Table 1:** The effect of pH on the porosity of silica nanoparticles under acid influence [44].

Acid Type	Gelation pH	Density (g cm^−3^)	Porosity (%)
Nitric Acid	4	0.849	61.23
	7	0.294	86.58
	9	0.273	87.53
Citric Acid	4	0.751	65.71
	7	0.432	80.27
	9	0.277	87.35

**Table 2:** The table indicates the effect of NH_4_OH on silica nanoparticles under the control condition of TOES, C_2_H_5_OH, and distilled water [50].

Sample	TOES (mL)	C_2_H_5_OH (mL)	DI Water (mL)	NH_4_OH (mL)	Average Particle Size (nm)
A	6.9	15	2.2	1.5	214.1
B	6.9	15	2.2	1.0	162.4
C	6.9	15	2.2	0.5	93.5

**Table 3:** The diagrammatic representation indicates the effect of silica concentration on total pore volume, BET surface area, and average pore width [60].

Sample	BET Surface Area (S_BET_, m^2^/g)	Total Pore Volume (V_b_, cm^3^ g^−1^)	Average Pore Width (r, Å)
Silica-25	596.61	0.3498	1.47
Silica-60	451.04	0.3247	1.80
Silica-100	338.32	0.2476	1.83
Silica-200	338.32	0.0466	1.83
Clay–silica core–shell	79.59	0.0763	2.40
